# Electrospun and co-electrospun biopolymer nanofibers for skin wounds on diabetic patients: an overview

**DOI:** 10.1039/d1ra02986j

**Published:** 2021-04-23

**Authors:** Paola I. Campa-Siqueiros, Tomás J. Madera-Santana, María M. Castillo-Ortega, Jaime López-Cervantes, Jesús F. Ayala-Zavala, Elizabeth L. Ortiz-Vazquez

**Affiliations:** Centro de Investigación en Alimentación y Desarrollo 83304 Hermosillo Sonora Mexico madera@ciad.mx; Universidad de Sonora 83000 Hermosillo Sonora Mexico; Instituto Tecnológico de Sonora 85000 Ciudad Obregón Sonora Mexico; Instituto Tecnológico de Mérida 97118 Mérida Yucatán Mexico

## Abstract

Wound healing treatment in diabetic patients worldwide represents around 2.1 trillion dollars to global health sectors. This is because of the complications presented in the wound healing process of skin ulcers, such as a lack of macrophage and fibroblast growth factors (TGF-β1 and PDGF, respectively) that are both needed for extracellular matrix (ECM) synthesis. Therefore, there is a need for research on new and cost-effective materials to enable ECM synthesis. Such materials include co-electrospun nanofibers used as wound dressings, since they have a similar morphology to the ECM, and therefore, possess the advantage of using different materials to accelerate the wound healing process. Co-electrospun nanofibers have a unique structural configuration, formed by a core and a shell. This configuration allows the protection and gradual liberation of healing agent compounds, which could be included in the core. Some of the materials used in nanofibers are polymers, including natural compounds, such as chitosan (which has been proven to possess antimicrobial and therapeutic activity) and gelatin (for its cell growth, adhesion, and organisational capacity in the wound healing process). Synthetics such as polyvinyl-alcohol (PVA) (mainly as a co-spinning agent to chitosan) can also be used. Another bioactive compound that can be used to enhance the wound healing process is eugenol, a terpenoid present in different medicinal plant tissues that have scarring properties. Therefore, the present review analyses the potential use of co-electrospun nanofibers, with chitosan–PVA–eugenol in the core and gelatin in the shell as a wound dressing for diabetic skin ulcers.

## Abstract 2

Wound healing treatment in diabetic patients worldwide represents around 2.1 trillion dollars to global health sectors.

## Graphical abstract

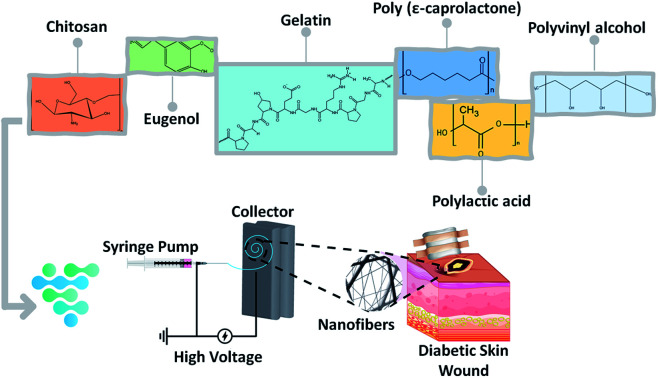

## 1. Introduction

The International Diabetes foundation reported an increase in the global diabetes prevalence from 151 million in 2000 to 463 million in 2019. It is predicted that the prevalence will increase to 578 million by 2030. This global increase would translate to a global economic burden of up to 2.1 trillion dollars upon health sectors for the treatment of derived complications from the disease, above all, skin wounds.^1^ The treatment cost for skin wounds, the complexity of the wound healing process, and complications for diabetic patients are among the reasons for the treatment costs. Moreover, the natural healing process is compromised in diabetic patients. In the second phase of the process (inflammation), platelet and macrophage growth factors (PDGF and TGF-β1 respectively), together with chemokines and cytokines, do not generally act on their cellular receptors. This lack of interaction translates into an absence of signalling cascades, jeopardising the cell proliferation, migration, and differentiation, which stops the wound healing process and could lead to amputation or even the death of the patient.^2^

To overcome the problems described, there is a need for research on new materials that can help the wound healing process for diabetic patients. Escárcega-Galaz *et al.*^3^ proposed a chitosan hydrogel for skin ulcers on diabetic feet (skin ulcer in the foot), and applied the treatment once every two days for three months. At the end of the study, the patients showed considerable healing of the wounds. A downside of the hydrogel treatment was that application could be messy, and there may be alternative materials that can accelerate the healing process.

Nanofibers obtained by electrospinning are a good alternative considering their versatile formulation (they can work with a large number of polymers) and the use of a cheap and simple processing technique. Another advantage of nanofibers is the increase in coverage area that can be obtained; this can be related to a higher healing power in comparison to fibres on a normal scale. Nevertheless, there are drawbacks to the electrospinning process and a high number of parameters can influence the final product, including the solution, process conditions, and environmental conditions. However, once the appropriate parameters are reached, the nanofiber production can progress smoothly.^4^

One variant of the electrospinning process is co-electrospinning, which has the same principles and works under the same parameters. The only difference is that with co-electrospinning, the resulting nanofibers have a more functional core–shell structure. This conformation enhances the processed materials, with improved healing properties.^5^

The reviewed evidence indicates that the more promising materials are gelatine for the shell fraction, and chitosan–PVA, and eugenol for the core fraction. Gelatine and chitosan are highlighted because of their biopolymeric nature, as well as their therapeutic properties.^6,7^ PVA was selected for material reinforcement and because of its good interaction with chitosan.^8^ Eugenol is a natural terpenoid with immune-modulatory activity as evidenced by Vishteh *et al.*^9^ more than 30 years ago.

In this context, the present review analyses the potential of co-electrospun nanofibres, with gelatine for the shell fraction and chitosan–PVA–eugenol in the core as a treatment for diabetic wounds.

## 2. Electrospinning

Among the techniques for nanofibre production, electrospinning is the one with the most advantages. It produces nanofibers at the laboratory or industrial level, with considerable reproducibility and efficiency. Moreover, it enables control of the nanofiber dimensions and great versatility in the use of polymeric materials.^4^ The resulting nanofibres have properties that give great application spectra in the biomedical field: high surface area to volume ratio and high porosity.^10^ There is evidence of their application as scaffolds for tissue regeneration,^11^ cartilage,^12^ bone,^13^ drug delivery,^14^ and wound dressings.^15^

Each application has been made with a variety of materials, specifically relating to the polymer–solvent mixture. The most important properties for choosing the adequate material is high molecular weight, as this allows entanglements that form the polymeric chains, and solubility.^16^Table 1 represents the different polymeric materials (either natural or synthetic) along with the most used solvent for nanofibre production. The present review is focussed on gelatine, PVA, and chitosan, with their respective solvents.

**Table 1:** The most used polymer and solvent blends for electrospun nanofibres^a^

Polymer	Solvent	Reference
Polyvinyl alcohol (PVA)	Water	17
Polycaprolactone (PCL)	Dimethyl formamide	18
Polyethylene (PE)	Melt	19
Polyethylene oxide (PEO)	Water	17
Polylactic acid (PLA)	Dimethyl formamide	20
Gelatin	Water, glacial acetic acid	21
Chitosan	Trifluoroacetic acid, glacial acetic acid	17

a Modified from Mitchell.^16^

### 2.1. Equipment

**Fig. 1:**
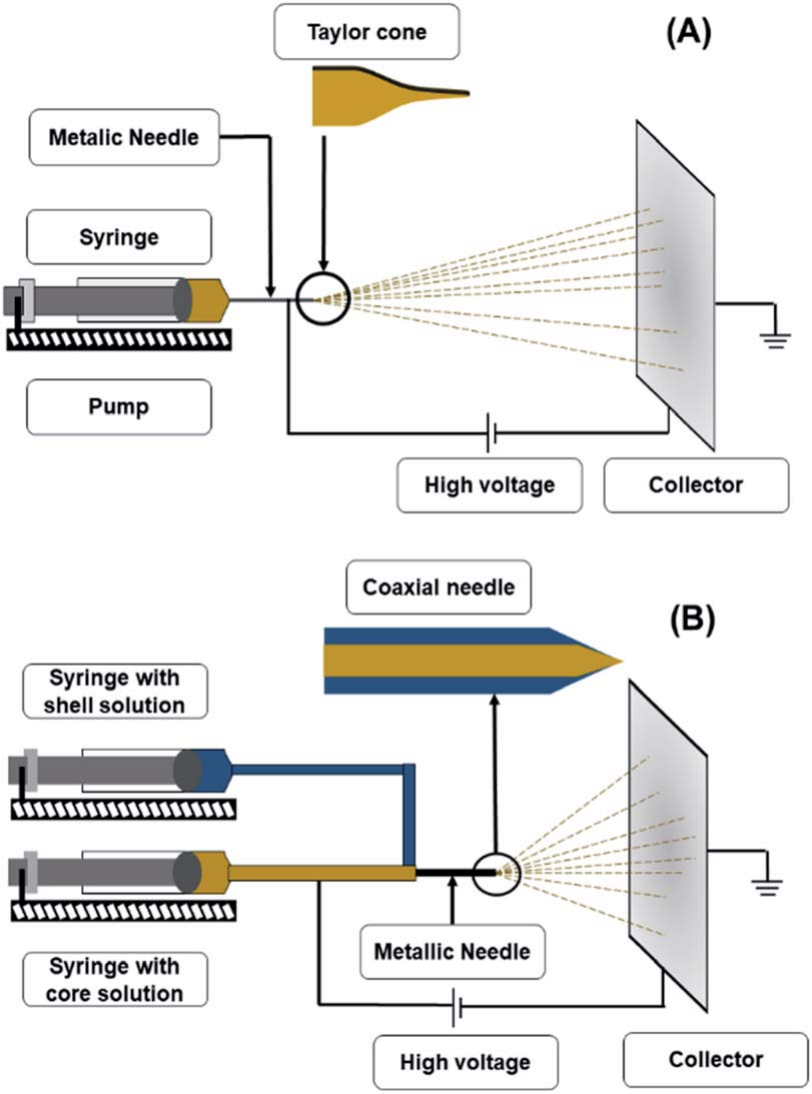
Schematic representation of electrospinning apparatus (A) and coaxial electrospinning apparatus (B).

The electrospinning apparatus (Fig. 1A) consists of four main components: a syringe with a metallic needle, a pump, a high voltage power supply, and a collector, which is generally earthed.^5,16^ The syringe supplies the polymeric solution at a rate established in the pump.^22^ The collector could be a fixed aluminium foil, or a rotating mandrel.^23^ The selection of the collector depends on the desired fibre alignment, since a metallic sheet produces randomly aligned fibres, whereas a rotating mandrel produces aligned fibers.^24^ Finally, the voltage power source provides a charge to the polymeric solution so that it can start its trajectory towards the collector with the opposite charge.^23^

### 2.2. Principle

**Fig. 2:**
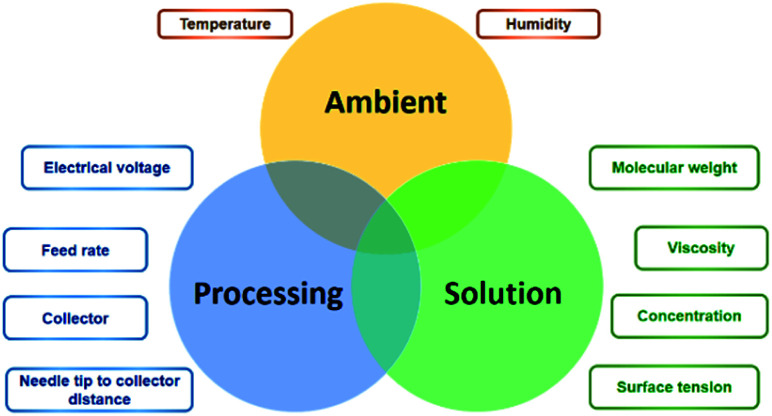
Main electrospinning factors and parameters.

In theory, the electrospinning technique consists of applying an electrostatic force (resulting in an electric field) using a high voltage supply previously described.^25^ The process is as follows: a droplet of the polymeric solution forms on the needle tip of the syringe. As the electric field increases, electrostatic charges start to concentrate upon the droplet. This produces a charge repulsion, which acts against surface tension, changing the droplet form to an elongated conical shape, known as a Taylor cone.^26^ As the field strength increases, the repulsive electrostatic force overcomes the surface tension and a charged jet of the polymeric solution starts to flow from the Taylor cone's tip towards the collector.^27^ The jet is a summary of the instabilities produced by the electric charges.^28^ Then, when the viscosity of the solution is defeated by the attraction forces, it produces a thread as a result of the materials' motion and stretching. Finally, the solvent evaporates, and the fibres solidify on the collector,^28^ with nano- to micrometric diameters.^27^ The electrospinning is considered a simple technique; however, it is a complex procedure. The many parameters influencing this process are generally divided into three categories: solution, processing, and ambient conditions (Fig. 2), which must come together to form nanofibers with the desired diameter, morphology, and porosity.^29^

### 2.3. Solution

#### 2.3.1. Molecular weight (*M*_w_)

A polymeric solution with high *M*_w_ produces nanofibers with larger diameters, whereas a low *M*_w_ polymer does not form fibres, but beads instead. An important characteristic of this parameter is its relation to the viscosity and the polymer chain entanglements.^23^ This behaviour was recently tested by Tian *et al.*,^30^ where they used polycaprolactone (PCL) with an average *M*_w_ of 80 000 g mol^−1^ at a concentration of 15% w/v and diluted it with a mixture of chloroform and ethanol, where the chloroform ratio was constant. In contrast, ethanol was varied from 1.9 : 1, 1.4 : 1, 1 : 1, 0.5 : 1, 0.3 : 1, and 0.1 : 1. For the electrospinning process, they used a flow rate of 400 μL h^−1^, a positive voltage of 10 kV, a distance of 15 cm between the needle and collector, and a volume of 2 mL. These authors directed their assay towards the polymer crystallisation; they studied the entanglement behaviour of the material. By using differential scanning calorimetry (DCS), they concluded that *M*_w_ is strongly associated with recovery of entanglement.

#### 2.3.2. Surface tension (ST)

High ST creates instability in the jet, which could disrupt the process and deliver droplets and beads instead of fibers.^31^ Low ST helps the process as long as a low electric field is used.^32^ It is important to clarify that ST is a function of the solvent used in the polymeric solution.^23^ Fortunately, there are different ways to change the ST of the materials used, by using additives, like ionic liquids or organic/inorganic salts, while varying the solvent or the surfactants.^23^

#### 2.3.3. Viscosity (*η*)

Unlike the other parameters, *η* is not directly or indirectly proportional to the nanofiber diameter. In this case, when the solution has a very high *η*, the jet cannot form. On the other hand, if *η* is very low, there is a jet ejection, but no fibre formation occurs. Hence the need to research the optimal *η* of the polymer solution. *η* basically determines the concentration range of the polymeric solution to obtain uniform and continuous nanofibres^26^ Tiwari and Venkatraman^33^ confirmed the effect of *η* on simple or core-shelled electrospun fibres, using a copolymer of polylactic acid (PLA) and polygalacturonic acid (PGA) in an 80 : 20 ratio, with chloroform and *N*,*N*-dimethylformamide (DMF) as the solvent. These authors measured the solution viscosities and plotted them against the solution concentration so that they could prove the *η*-concentration dependency. For a better understanding, this relationship will be discussed with the following parameter.

#### 2.3.4. Concentration (Co)

As explained before, this parameter is intimately correlated with *η*. If we have a polymeric solution with low concentration, there is not just fibre formation, but the presence of beads as well. If we increase the concentration, the shape changes to uniform fibres followed by an increased diameter, and this is a reason for a higher *η* resistance. There are four concentration regimes for polymer solutions: 1. concentrated, 2. semidilute entangled, 3. semidilute untangled, and 4. dilute. According to the study of Tiwari and Venkatraman,^33^ they reported an especially important concept, an overlap in concentration (C*). Its importance lies in the polymeric chain behaviour since it is in this concentration, where the individual chains that were separated from the solvent at the dilution start overlapping with each other; however, they remain untangled. As the concentration increases, the solution starts developing entanglements. This is a result of the induced topological constraints formed because of the larger occupied fraction of the available hydrodynamic volume in the solution.

#### 2.3.5. Conductivity (*C*)

Concerning the nanofiber diameter, as the polymeric solution's electrical conductivity increases, the fibre diameter decreases. This indirect proportionality is because of the incapability of the electrical force to produce an insufficient elongation of the jet.^23^

### 2.4. Processing

#### 2.4.1. Feed rate (FR)

A low FR is desired during the process since it allows for complete solvent evaporation. On the other hand, high FR values do not allow for solvent evaporation, which causes the formation of beads. Therefore, FR is directly proportional to the fibre diameter. This was proven by Someswararo *et al.*^34^ since they observed an increased diameter from 111 nm to 214 nm on TiO_2_ nanofibers when varying the FR from 0.6 mL h^−1^ to 1.2 mL h^−1^. Also, this parameter is important because it influences the material transfer rate and jet velocity.

#### 2.4.2. Electrical voltage (*V*)

This parameter is probably the most important in the whole electrospinning process. As discussed before, for an electrospinning process to occur, an electrical force must be applied to the material. In this sense, the relationship between *V* and diameter is inversely proportional. Yuan *et al.*^35^ analysed the morphology of electrospun fibres of polysulfone/DMAC/acetone. They observed that an increase in voltage caused a decrease in the fibre diameter. They concluded that the phenomenon was a consequence of an increase of the electrostatic repulsive forces of the charged jet that resulted from the voltage increase. This effect on the jet leads to smaller diameters.

#### 2.4.3. Needle tip to collector distance (CD)

The objective of this parameter is to give the jet enough time for the solvent to evaporate and the nanofiber to solidify on the collector. Dhandayuthapani *et al.*^36^ studied the morphology of electrospun nanofibers of gelatine and chitosan, starting at a distance range of 5 cm to 15 cm with the other parameter's constant. They found that 5 cm was too small to either maintain a stable jet or have enough solvent volatilisation. Nevertheless, they obtained good nanofibers at 10 cm and 15 cm, and at 15 cm, the fibre density collected was low. The authors concluded that the effect was a consequence of the formation of smaller fibre jets due to the bending instabilities, and these could not reach the collector.

#### 2.4.4. Collector (Coll)

The collector in electrospinning is the conductive substrate to collect the nanofibers.^23^ It has an effect on the alignment instead of affecting the fibre morphology. This alignment depends on the collector and the rotation speed. In the case of a rotating collector, the resulting nanofibers should have a certain order grade. This is contrary to the fixed grounded collector, whereby the bending instability of the charged jet causes the random deposition of the nanofibers.^31^

### 2.5. Ambient

#### 2.5.1. Temperature

The first environmental parameter to consider in an electrospinning process is the temperature. With the more basic equipment, the process is normally non-adiabatic and it is difficult to control the heat transfer. This affects *η*; therefore, its increment is inversely proportional to the nanofiber diameter. This behaviour was confirmed by Mit-uppatham *et al.*,^37^ who spun polyamide-6 nanofibers and observed that their diameter decreased with increasing temperatures.

#### 2.5.2. Relative humidity (RH)

RH is perhaps the most difficult parameter to control in the basic equipment. High humidity results from the presence of free volume on the nanofibers. This effect was reported by Casper *et al.*^38^ since they evaluated the RH effect on polystyrene nanofibers. They managed a range of 31–38%, 40–45%, 50–59%, 66–72%, and observed that the range of pore diameter increased proportionally with the RH values.

## 3. Co-electrospinning

Also known as coaxial electrospinning, co-electrospinning is a modification of the traditional electrospinning technique (Fig. 1B). The same principle and parameters apply; only in this case, instead of one needle for the polymeric solution, there are two needles connected to two different polymeric solutions.^39^ The needle conformation gives a unique nanofiber conformation known as the core–shell. This process can have the same applications of electrospinning; however, the nanofiber conformation provides extra protection to any compound included in the core. Another advantage of this technique is that the trapped compound is protected from environmental stress and its release can be controlled.^40,41^

There are reports of core–shell nanofibers used for biomedical purposes, such as a study by Jalaja *et al.*^42^ These authors produced co-electrospun nanofibers' with chitosan as the shell material, gelatine as the core, and aqueous acetic acid solutions as the solvent. To enhance the nanofibers water resistance, oxidised dextran and sucrose were used as cross-linkers, and the resulting material was biologically evaluated with human osteoblast-like cells (MG-63 cells). The treated cells grew at a normal rate in the nanofiber mats. They concluded that this favourable result was closely related to the ability of core–shell nanofibers to enhance the biological activities of both chitosan and gelatine. The enhanced cell attachment and proliferation resulted from the presence of gelatine in the core and chitosan on the surface of the nanofibers.

## 4. Biopolymers for electrospun nanofibers

Biopolymers are one of the most frequently used materials for electrospun nanofibers with a biomedical application. By definition, a biopolymer involves large macromolecules formed by covalent bonded monomers, which can be broken into smaller chains by the action of biological factors. Generally, biopolymers are classified based on their origin, that is to say, natural or synthetic.^43^

Natural biopolymers possess various properties of interest for biomedical application. Apart from being biodegradable and biocompatible, natural biopolymers present biological recognition, impacting positively upon cell function and adhesion.^44^ In the case of nanofibers for wound healing, some of the most reported natural biopolymers assayed are gelatine and chitosan. However, their mechanical properties are limited, hence the need to complement them with other materials.^45^ Synthetic biopolymers on the other hand, are recognized by their robust mechanical properties, and have a reduced production cost in comparison to natural biopolymers.^46^ Among the most assayed synthetic biopolymers as nanofibers for wound healing are PLA, PCL, and PVA.

### 4.1. Gelatine

**Fig. 3:**
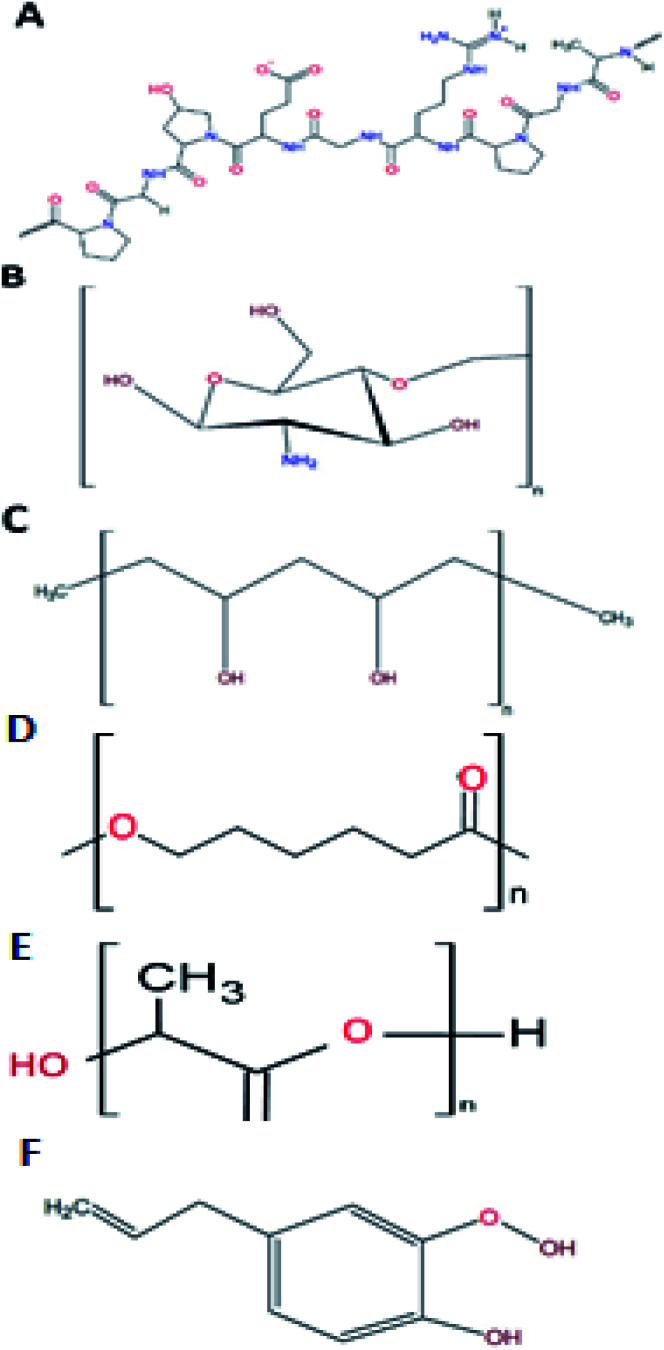
Typical structure of gelatine (A), chitosan (B), PVA (C), PCL (D), PLA (E), and eugenol (F).

As addressed before, gelatine is a promising material to be used in the shell phase of the co-electrospun nanofibers. This biopolymer is a protein (Fig. 3A) that results from either the acid (type A) or alkaline (type B) hydrolysis of collagen, with 300 to 4000 amino acids.^6^ From this composition, 25% is a combination of positive and negative charged residues (lysine and arginine, and aspartate and glutamate, respectively), 11% are hydrophobic (methionine, leucine, isoleucine, and valine), and the remaining percentage consists of proline, hydroxyproline, and glycine.^36^

Aramwit *et al.*^47^ made a comparison among nanoparticles of different gelatine types compounds. The authors used models for positive and negative charges using methylene blue and eosin respectively, and sericin as a biologically active compound. The difference between the responses of each gelatine type can be explained as follows. The differences among the crosslinking degree could be a consequence of the added glutaraldehyde. This agent binds the aldehyde groups with the amine groups of gelatine. Since type B gelatine has a higher amount of free amine groups, it caused a higher crosslinking degree. Sericin is an active compound with a negative charge, which in this study was entrapped upon a gelatine type B nanoparticle also negatively charged.

As a law, equal charges tend to repel, an effect not seen in this case. Therefore, the authors proposed that perhaps electrostatic interaction is not always the main mechanism, but forces such as van der Waals interactions and hydrogen bonding could also take place on the release behaviour of molecules from carriers.

The property of gelatine to release bioactive molecules highlights the biomedical importance of this polymer. For example, its uses in adhesive and scaffolds treatments have been previously reported, along with macrophage stimulation^48^ and hemostatic effect.^49^ This behaviour was reported by Dubsky *et al.*,^50^ where they assayed electrospun nanofibers of PCL, gelatine, and cotton as a control, observing that either on day five or 10, gelatine was the compound with the higher wound closing percentage. Thus, gelatine can be postulated as a reliable compound for its use in biomedicine, especially gelatine B. It shares characteristics with chitosan.

### 4.2. Chitosan

Chitosan is the product of the hydrolysis of chitin, the second most abundant natural polymer.^7^ Chitin, with a degree of deacetylation higher than 60%, is considered as chitosan.^49^ Chitosan's structure is formed by two monomers, *N*-glucosamine and *N*-acetyl-d-glucosamine, linked by a β (1–4) bond^51^ with an amine group and two hydroxyls as its active groups^7^ (Fig. 3B). Its amine groups are the ones that provide chitosan with its unique characteristics. At a pH lower than 6, chitosan's amine groups acquire a positive charge, making the interaction between this group and negatively charged metallic ions (Cu, Mg, and Fe) and biomolecules (fatty acids, phospholipids, anionic polysaccharides, and proteins) possible.^51^

The chitosan properties mentioned above warrant its biomedical importance, with diverse uses in drug release systems,^52^ tissue engineering,^53^ and wound healing^54^ For this purpose, chitosan is almost the perfect candidate to form nanofibres, since it has antimicrobial and therapeutic activities, which will be explained as follows.

#### 4.2.1. Antimicrobial activity

**Fig. 4:**
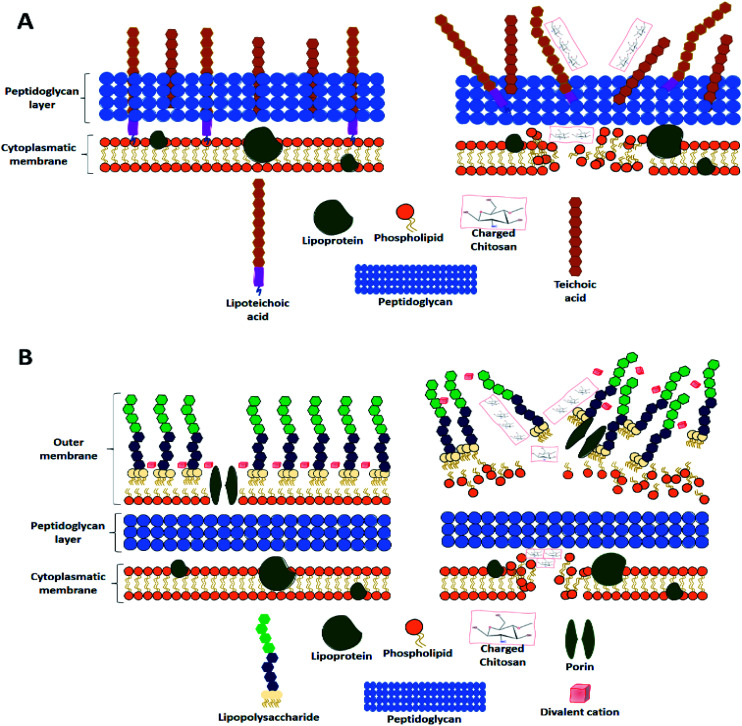
Antibacterial activity of chitosan upon (A) Gram positive and (B) Gram negative bacteria.

There are four proposed antimicrobial mechanisms for chitosan, against both Gram-positive (Fig. 4A) and Gram-negative bacteria (Fig. 4B).^55^

The first mechanism is related to a chitosan characteristic previously mentioned, its capacity to interact and chelate negatively charged metal ions. The interaction produces a cell wall disruption, which damages the microorganism's integrity.^56^ However, for this mechanism to take place, chitosan has to be at a pH above its p*K*a (6.3–6.5), so its amino groups can be protonated.^57^ The second mechanism is the occurrence of electrostatic interactions; this mechanism applies to Gram-negative and Gram-positive bacteria despite their different membrane components. Upon contact with Gram-negative bacteria, the electrostatic interactions take place between the cationic chitosan and the anionic lipopolysaccharide present in the outer membrane.^58^

The third mechanism involves the electrostatic interactions between chitosan and one of the most important components of the peptidoglycan layer in the cell surface, teichoic acid.^59^ This acid has three key functions in the cell membrane: control of enzymatic activity and cationic concentration, environmental stress protection, and serving as an anchor of the cell to surfaces.^60^ Therefore, an interaction between chitosan and the teichoic acids quite possibly results in multifactorial cell death.

Finally, the diffusion mechanism of chitosan within the bacterial cell depends on its molecular weight more than its charge properties. At a certain weight, chitosan can permeate the membrane of Gram-negative bacteria, possibly damaging the DNA/RNA synthesis, and their survival.^61^

#### 4.2.2. Wound healing properties

Wound healing is a complex process in which a great number of different molecules are involved, such as the extracellular matrix (ECM). Therefore, it is imperative that we review the differences in the wound healing process between a healthy person and a diabetic patient.

The wound healing process could be divided into four phases: haemostasis, inflammation, proliferation, and remodelling.^6^ In a review article, Liu *et al.*^58^ explained the way chitosan (as a hydrogel) participates in the wound healing stages. In summary, the most important action of chitosan is to promote platelet activation.

Platelets are essential components of the coagulation process since they release cytokines. In the inflammatory phase, the cytokines along with growth factors and chemokines, activate an intracellular signalling cascade, giving a place for cellular proliferation, migration, and differentiation, along with the recruiting of granulocytes and macrophages. This phase is when the difference between the wound healing process of a healthy person and a diabetic person starts to appear. In a healthy person, there is the presence of a platelet-derived growth factor (PDGF) and a transforming growth factor (TGF)-β1, both released from the platelets. However, a diabetic person has a deficient expression of these factors. This adds to the fact that the inflammatory phase is the foundation for the process. But, if it is present in excess, it could lead to tissue necrosis and systemic infection, which are known symptoms present upon the wounds of diabetic patients.^62^

Okamoto *et al.*^44^ reported that chitosan was able to provide two very important features in the wound healing process for a diabetic person: it could induce the release of PDGF and (TGF)-β1, activating macrophage and fibroblast proliferation, thereby promoting cytokines and collagen synthesis, respectively. Chitosan also, provides a 3D matrix for tissue growth. If it is not enough, chitosan depolymerises to *N*-acetyl-glucosamine, one of the major components of dermal tissue essential for scar tissue repair.^63^ Moreover, this depolymerisation increases hyaluronic acid and collagen type III synthesis.^64^

Despite having all the advantages presented above, chitosan shows some disadvantages. Its ionic nature, demonstrated by the protonated amines, makes the production of pure chitosan nanofibers rather difficult. This ionic nature of chitosan provides a high superficial tension^65^ that, according to the electrospinning parameters previously addressed, results in the formation of droplets or beads instead of fibers.^59^ Therefore, chitosan needs an electrospinning agent to be able to form nanofibers with good characteristics. Some of these agents are PLA, PCL and PVA.^23^

### 4.3. Polyvinyl alcohol

Polyvinyl alcohol (PVA) is a synthetic biopolymer that is highly soluble in water. Compared to other polymers with vinyl groups, PVA is obtained by hydrolysis of polyvinyl acetate (PVAc) in an alcohol solution, instead of a polymerisation process. Structurally, PVA is an atactic linear polymer, with hydroxyl groups randomly positioned along the chain (Fig. 3C). An essential property of PVA to predict its behaviour in solution is the hydrolysed percentage of the polymer. There are three hydrolysis levels: partial, medium, and full. This classification depends upon the molar percentage of the remaining acetate groups. Since it directly affects the solution viscosity, it affects the electrospun nanofiber formation (as discussed with the electrospinning parameters). In this case, the relationship between the hydrolysis percentage and *η* is directly proportional.^8^

Concerning the use of PVA in biomedicine, the type of material most reported for this end are hydrogels. PVA is used as a therapeutic agent based on its good biocompatibility.^66^ Recently, Alavarse *et al.*^29^ assayed the possibility of using chitosan–PVA electrospun nanofibers as a delivery system for tetracycline hydrochloride. These authors reported that pure PVA nanofibers have good mechanical properties. However, its high hydrophilicity results in a high rate of biodegradation due to the hydroxyl groups. This behaviour is considered a disadvantage if the purpose of the material is for a wound dressing. Yet when combined with chitosan the degradation rate decreases, since inter and intramolecular hydrogen bonds between chitosan and PVA enhance the mechanical properties of the electrospun nanofibers. Therefore, the combination of these biopolymers would result in a win–win situation for obtaining electrospun nanofibers for wound dressings.

Another recent report of the use of chitosan/PVA blend electrospun nanofibers for therapeutic use is the study by Sedghi *et al.*^67^ However, in this case, a natural active compound (curcumin) was used.^29^ These authors reported that when the PVA concentration decreases, the repulsive forces of chitosan amino groups might interfere with an effective chain entanglement. Therefore, with this type of blend, a high PVA concentration is recommended to obtain beadless electrospun nanofibers. Additionally, reported in this study was the importance of the solvent in electrospun nanofiber processing. Because acetic acid has a higher vapour pressure compared to water, as the PVA concentration increased, the water to acetic acid ratio in chitosan–PVA–curcumin solution decreased gradually. Therefore, if the electrospinning parameters apply, this easy acetic acid evaporation resulted in a faster polymeric jet solidification and good nanofiber morphology.

There are numerous assays which have proved the possible use of different biopolymers as a wound healing scaffold, specifically on diabetic patients. Table 2 summarises a series of articles that have proven with *in vivo* assays on mice that electrospun nanofibers possess the necessary biocompatibility, adhesion, and lack of cytotoxicity to carry on with this application. Also present in Table 2 is the common practice of using an active compound to enhance the wound healing properties of the electrospun nanofibers.

**Table 2:** *In vivo* assays of electrospun nanofibres with wound healing application on diabetes induced mice

Technique	Polymer	Active compound	Reference
Electrospinning	PCL/gum tragacanth (GT)	Curcumin	68
Electrospinning	PCL and PEG	Epidermal growth factor (EGF)	69
Electrospinning	PCL and gelatin	Endothelial progenitor cells	70
Electrospinning	Chitosan–PVA	Nanobioglass	71
Electrospinning (aligned)	PLGA	Curcumin and heparin	72
Electrospinning	Poly(3-2 hydroxybutyrate-*co*-3-hydroxyvalerate) (PHBV)	Epidermal growth factor (EGF)	73
Electrospinning	GT, PCL, and PVA		74
Liquid-collecting electrospinning	PLGA	Collagen type I	75
Spraying, phase-inversion, and electrospinning	Fibrin/poly(ether) urethane	Platelet lysate (PL)	76

### 4.4. Polycaprolactone

Polycaprolactone (PCL) is a synthetic biopolymer from the family of polyesters.^77^ This biopolymer (Fig. 3D) is approved by the Food and Drug Administration (FDA). It is aliphatic and semi-crystalline in nature, with a melting temperature range of 59–34 °C and a glass transition temperature of −60 °C, represented as high toughness.^78^ One of the most interesting properties of PCL for wound healing application resides on its degradation time. Compared to other polyesters, PCL presents a degradation time of 2 to 3 years, either by hydrolysis of its aliphatic ester bond or by microorganisms.^79,80^

Drug delivery, bone regeneration and wound healing are among the different biomedical applications with PCL that have been examined, however, the major obstacle for this application is the high hydrophobicity present on PCL. Therefore, there is a need to complement the materials with other polymers to counteract the effect, such as chitosan.^81^ Ho *et al.*^82^ fabricated electrospun nanofibers coated with chitosan oligomers (COS) for wound healing application. Their results showed that their materials presented a high bacterial inhibitory activity and biocompatibility, so long as COS did not exceed a certain concentration. Also, COS concentration affects reepithelization and wound healing in mice, with the samples of 15% w/v PCL and 8% w/v COS presenting the best *in vivo* performance. These authors bestow this activity to the free radical scavenging activity of COS in the wound site.

Ranjbar-Mohammadi *et al.*^68^ assayed the antimicrobial and *in vivo* activity on diabetic mice of PCL and gum tragacanth (GT) loaded with curcumin (Cur). These authors reported that their materials presented an effect on increasing the collagen content upon diabetic wounds, promoting healing, particularly in the early stages. These nanofibers presented a synergy between the GT high biological properties, PCL high physical–mechanical properties and the sustained release of Cur (a reactive oxygen species reductor), resulting in stable scaffolds in front of blood and fibrin as well.

### 4.5. Polylactic acid

Another polyester of importance in diabetes wound healing is polylactic acid (PLA). This biopolymer (Fig. 3E) has the peculiarity that the main precursor, lactic acid, is produced in large amounts as result of carbon source fermentation by lactic acid bacteria, such as *Lactobacilli*.^6^ Lactic acid is the product of the fermentation of dextrose, derived from plant starch. This means that PLA is a biopolymer that could reduce the dependence on fossil-based resources for plastic obtention.^83^ PLA has a glass transition temperature range from 40 to 70 °C and a melting temperature between 130 to 180 °C, with a tensile strength of 440 to 59 MPa. These characteristics make PLA a hydrophobic semicrystalline polymer.^6^

PLA possesses outstanding biodegradability and biocompatibility, making it a great material for regenerative medicine, gene transfer, drug delivery and tissue engineering.^84^ Fang *et al.*^85^ prepared core–shell nanofibers with PLA as the shell and γ-PGA as the core by electrospinning for wound healing application. These authors reported beneficial cell proliferation and good biocompatibility, reflected as restoration of epidermal and dermal tissue on mice after 14 days.

There have also been reports of PLA electrospun nanofibers for wound healing on diabetes induced mice. Han *et al.*^86^ fabricated aligned electrospun nanofibers from PLA and asiatic acid (AA) for wound healing acceleration. Also, they reported that their materials, particularly the samples with 30% AA, presented excellent accelerating reepithelization, ECM formation and angiogenesis *in vivo* in diabetic mice. These authors confer this behaviour to the AA effect on anti-oxidative stress, anti-bacteria, and anti-inflammation *in vitro*. Also, PLA aligned nanofibers promoted wound healing acceleration by facilitating fibroblasts and keratinocytes migration from the periphery to the centre of the wound, therefore promoting collagen formation and reepithelization.

The literature indicates that, although a natural bioactive compound is present on the nanofibers, it is possible to obtain electrospun nanofibers with good characteristics. As such, other natural bioactive compounds could be used as well.

### 4.6. Eugenol

Belonging to the phenylpropene family, eugenol is structurally composed of an aromatic phenyl group and a propane tail (Fig. 3F), produced in the first step of the phenylpropanoid biosynthesis.^87^ Eugenol can be extracted from different plants, such as clove (*Eugenia caryophyllata*) and basil (*Ocimum basilicum* L.),^88^ and one of the best solvents for its extraction is glacial acetic acid.^89^ Several properties have been attributed to eugenol, such as antioxidant, antispasmodic, pharmaceutical, anti-inflammatory, antimicrobial, anaesthetic, and antiseptic. Also, it is recognised as generally safe by the FDA.^88^ It is classified as a category 3 compound by the US Environmental Protection Agency. The oral LD_50_ is >1930 mg kg^−1^ in rodents.^88^ However, when used at an optimal dose, eugenol even enhances the immune system response against infectious agents and tumour cells.^90^

Nam and Kim^90^ studied the effect of the antioxidant and anti-inflammatory properties of eugenol in a metalloproteinase (MMP-9) metastasis matrix. These authors reported that eugenol presented a high inhibitory effect on hydrogen peroxide compared to other reactive oxygen species (ROS) and had the ability to block lipid peroxidation induced by hydroxyl radical and block DNA oxidation. Also, eugenol inhibits MMP-9 by inactivating ERK-1 *via* its antioxidant activity.

**Fig. 5:**
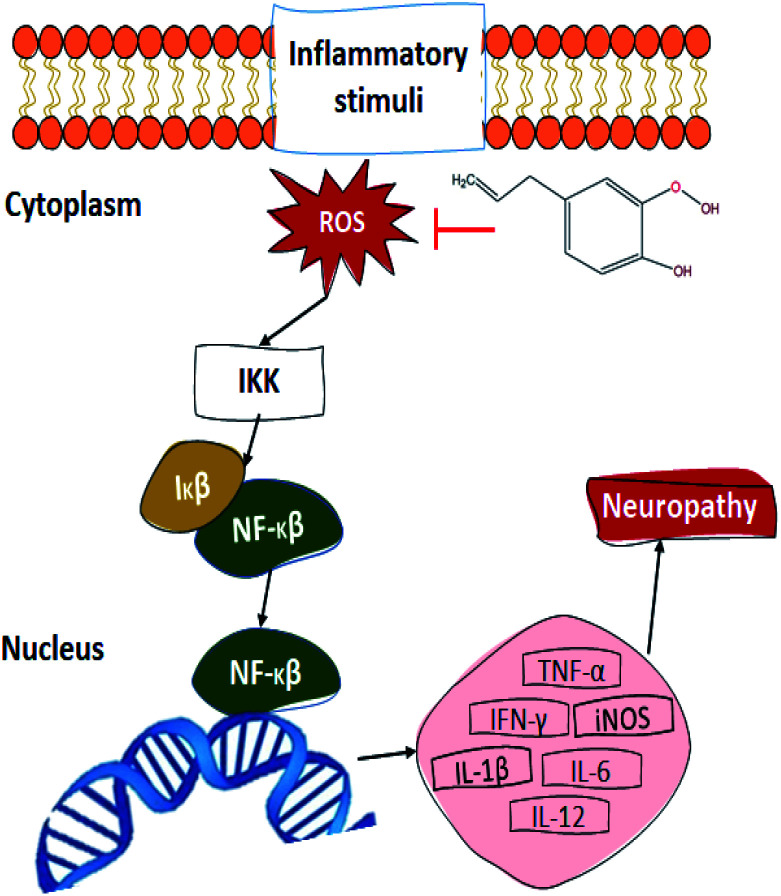
Proposed mechanism of eugenol upon NF-κβ pathway.

Another proposed mechanism (Fig. 5) through which eugenol could promote wound healing upon diabetic patients is the relation between its antioxidant property and the transcriptional nuclear factor beta (NF-κβ). In the presence of ROS, the inhibitor kappa beta kinase (IKK) activates, releasing NF-κβ and therefore promoting its translocation to the nucleus, activating between 200 and 300 genes. Some of the NF-κβ activated genes decode for pro-inflammatory cytokines, which is one of the reasons that diabetic patients present such slow wound healing. The tumour necrosis factor (TNF-α), interleukins (IL), induced nitric oxide synthase (iNOS), and interferon-gamma (IFN-γ) are some of the pro-inflammatory cytokines which become activated by the NF-κβ pathway. These cytokines contribute to the pathogenesis and maintenance of the usual neuropathy presented in diabetic patients, promoting nervous excitability and therefore, inducing pain.^91–93^ As for the antimicrobial activity of eugenol, there is a general idea about its action mechanism, which relates to the alteration of its permeability mechanism by damaging and altering lysosome, microsome, and cell walls. This damage causes bacterial death by the leakage of its essential cell constituents.^90^ Finally, the aesthetic and antiseptic properties of eugenol can be attributed to its molecular structure, since it contains *para*-allyl and *ortho*-methoxy groups.^89^

## 5. Conclusion

Electrospinning and co-electrospinning are simple, yet powerful processes which can create morphologically homogeneous nanofibers and can help cell growth and cell tissue regeneration. To summarise, if the materials conforming the nanofibers are biopolymers (either natural, synthetic or both), the resultant membrane would be a dressing with the necessary physicochemical properties to carry out their application. Furthermore, if the wound dressing is accompanied by a natural active compound, such as eugenol, the target of the wound dressing would be cantered to influence healing upon a specific type of wound, such as diabetic wounds.

## Conflicts of interest

The authors declare that there are no conflicts to declare.

## Supplementary Material
